# Structural Studies of a Bacterial tRNA^HIS^ Guanylyltransferase (Thg1)-Like Protein, with Nucleotide in the Activation and Nucleotidyl Transfer Sites

**DOI:** 10.1371/journal.pone.0067465

**Published:** 2013-07-03

**Authors:** Samantha J. Hyde, Bhalchandra S. Rao, Brian E. Eckenroth, Jane E. Jackman, Sylvie Doublié

**Affiliations:** 1 Department of Microbiology and Molecular Genetics, University of Vermont, Burlington, Vermont, United States of America; 2 Department of Chemistry and Biochemistry, Center for RNA Biology and Molecular, Cellular and Developmental Biology Graduate Program, The Ohio State University, Columbus, Ohio, United States of America; Institute of Enzymology of the Hungarian Academy of Science, Hungary

## Abstract

All nucleotide polymerases and transferases catalyze nucleotide addition in a 5′ to 3′ direction. In contrast, tRNA^His^ guanylyltransferase (Thg1) enzymes catalyze the unusual reverse addition (3′ to 5′) of nucleotides to polynucleotide substrates. In eukaryotes, Thg1 enzymes use the 3′–5′ addition activity to add G_−1_ to the 5′-end of tRNA^His^, a modification required for efficient aminoacylation of the tRNA by the histidyl-tRNA synthetase. Thg1-like proteins (TLPs) are found in Archaea, Bacteria, and mitochondria and are biochemically distinct from their eukaryotic Thg1 counterparts TLPs catalyze 5′-end repair of truncated tRNAs and act on a broad range of tRNA substrates instead of exhibiting strict specificity for tRNA^His^. Taken together, these data suggest that TLPs function in distinct biological pathways from the tRNA^His^ maturation pathway, perhaps in tRNA quality control. Here we present the first crystal structure of a TLP, from the gram-positive soil bacterium *Bacillus thuringiensis* (BtTLP). The enzyme is a tetramer like human THG1, with which it shares substantial structural similarity. Catalysis of the 3′–5′ reaction with 5′-monophosphorylated tRNA necessitates first an activation step, generating a 5′-adenylylated intermediate prior to a second nucleotidyl transfer step, in which a nucleotide is transferred to the tRNA 5′-end. Consistent with earlier characterization of human THG1, we observed distinct binding sites for the nucleotides involved in these two steps of activation and nucleotidyl transfer. A BtTLP complex with GTP reveals new interactions with the GTP nucleotide in the activation site that were not evident from the previously solved structure. Moreover, the BtTLP-ATP structure allows direct observation of ATP in the activation site for the first time. The BtTLP structural data, combined with kinetic analysis of selected variants, provide new insight into the role of key residues in the activation step.

## Introduction

The tRNA^His^ guanylyltransferase (Thg1) family comprises enzymes from all three domains of life, all of which catalyze reverse addition (3′–5′) of nucleotides to polynucleotide substrates [1]. The founding member of the Thg1 enzyme family, *Saccharomyces cerevisiae* Thg1 (ScThg1), adds a single highly conserved G residue (G_−1_) to the 5′-end of tRNA^His^ species using three chemical steps, all catalyzed by Thg1 [2]–[4]. In the first step, Thg1 uses ATP to activate the 5′-monophosphorylated tRNA^His^ that is generated by Ribonuclease P (RNase P)-catalyzed cleavage of pre-tRNA^His^, producing a 5′-adenylylated tRNA^His^ intermediate. In the second step, the 3′-hydroxyl of a GTP nucleotide attacks the activated intermediate, yielding the triphosphorylated form of tRNA^His^ (ppp G_−1_-tRNA^His^). In the third step, the mature (p)G_−1_-tRNA^His^ is produced by removal of the 5′-pyrophosphate.

Thg1 is essential in yeast, and presumably throughout eukaryotes, because the G_−1_ at the 5′ end of tRNA^His^ molecules is required for recognition of the tRNA by its cognate histidyl-tRNA synthetase (HisRS). In contrast the role of Thg1 family enzymes from other domains of life is less obvious [5]–[7]. Many bacterial and archaeal species that contain a Thg1-like protein (TLP) do not require post-transcriptional addition of the G_−1_ residue [8]. In these organisms, G_−1_ is instead encoded by the tRNA^His^ gene, incorporated into tRNA^His^ during transcription and retained in the tRNA after RNase P processing, obviating the need for TLPs in tRNA^His^ maturation [9]. Moreover, TLP enzymes differ from their eukaryotic counterparts because of their inability to form non-Watson-Crick base pairs during the 3′–5′ addition reaction, whereas the ability to incorporate a non-templated G_−1_ opposite a universally conserved A_73_ discriminator nucleotide in tRNA^His^ is a hallmark of eukaryotic Thg1-type enzyme activity. Instead, TLPs prefer to catalyze Watson-Crick template-dependent 3′–5′ reverse polymerization, suggesting that alternative roles for Thg1 family enzymes may exist that would take advantage of the ability to catalyze this unusual polymerase reaction, which occurs in the opposite direction to all other known DNA/RNA polymerases [10], [11].

One possible function for TLPs is suggested by the observation that bacterial and archaeal TLPs use template-dependent 3′–5′ polymerase activity to catalyze 5′-end repair of truncated tRNA species *in vitro* [12]. For the repair reaction, TLPs use the 3′-acceptor stem nucleotides as a template to restore a fully base paired aminoacyl-acceptor stem [1]. Moreover, unlike the G_−1_ addition to tRNA^His^ catalyzed by yeast Thg1, the repair function of TLPs is not limited to tRNA^His^, but occurs efficiently with other tRNAs [12]. This observation suggests that TLPs may play general roles in tRNA or other RNA quality control mechanisms. In the mitochondria of certain protozoan species, such as *Dictyostelium discoideum*, one or more TLPs have been implicated in an unusual 5′-tRNA editing reaction in which these enzymes use the tRNA 5′-end repair activity to replace encoded mismatched nucleotides with correctly base paired nucleotides in mature tRNAs [13]–[15].

Recently, the crystal structure of human THG1 (hTHG1), the first structure of any Thg1/TLP family enzyme, was solved, providing general insight into catalysis of the 3′–5′ addition reaction by members of this unusual enzyme family [16]. Intriguingly, the structure revealed that, despite a lack of identifiable sequence similarity between Thg1 and any other known enzyme family, hTHG1 shares remarkable structural similarity to canonical 5′–3′ DNA/RNA polymerases. The hTHG1 active site contains two metal ions coordinated by highly conserved carboxylate residues [16]. The two magnesium ions correspond to the two metal ions known to catalyze 5′–3′ nucleotide addition in traditional 5′–3′ polymerases [17]–[19]. These structural similarities combined with the results of alteration of several Thg1 active site residues suggest that Thg1 also uses a similar two metal-ion mechanism for 3′–5′ addition, raising questions about the evolution of 5′–3′ and 3′–5′ nucleotide addition activities in biology. The crystal structure of hTHG1 contains a bound dGTP nucleotide, which is suggested to adopt the position of the nucleotide used for the activation step of the 3′–5′ addition reaction [3]. However, important features of the Thg1 mechanism, including the mechanism of tRNA and NTP substrate-positioning for the formation of Watson-Crick vs. non-Watson-Crick base pairs during 3′–5′ addition, and the biochemical basis for the distinctions between the activities of Thg1 vs. TLPs remain unknown.

Here we report the first crystal structure of any TLP, that of the gram-positive soil bacterium *Bacillus thuringiensis* (BtTLP). Consistent with the hypothesis that all Thg1/TLP enzymes share the basic ability to use two metal-ion catalysis for template-dependent 3′–5′ nucleotide addition, BtTLP shares similar active site architecture to that seen in hTHG1. Alteration of active-site residues confirms that the highly conserved metal-coordinating carboxylates are similarly essential for activity of BtTLP and reveals the biochemical basis for participation of a highly conserved lysine residue in the activation step of catalysis. These results represent the first structural characterization of a Thg1/TLP family enzyme whose primary function is 5′-end repair, and are the foundation for understanding substrate selection by different Thg1/TLP family enzymes.

## Materials and Methods

### Protein Expression and Purification

BtTLP was produced by overexpression of the N-terminal (His)_6_ fusion protein in *Escherichia coli* Rosetta 2 (DE3) pLysS cells (Novagen). Cells were induced with 0.5 mM isopropyl-β-D-thiogalactoside and grown at 30°C for 12 h. Initial purification was performed using nickel-nitrilotriacetate (Ni-NTA) beads (Qiagen). Affinity chromatography was then performed using a HiTrap Blue HP column (GE Healthcare). Protein was eluted in a 100–1000 mM KCl gradient buffered in Tris-HCl, pH 7.5. Protein was then concentrated to 20–40 mg/mL (Millipore Amicon Ultra-15), flash frozen in liquid nitrogen and stored at −80°C.

### Crystallization

BtTLP orthorhombic crystals were obtained by vapor diffusion. Hanging drops were composed of 1 μl protein (2 mg/ml) and 1 μl reservoir solution (13% (w/v) polyethylene glycol 4000, 50 mM MgSO_4_ and 100 mM Tris(2-carboxyethyl) phosphine, pH 7) and ∼1 mM ATP. Crystals were transferred to a drop containing a cryoprotection solution made of either 50% (w/v) glycerol and 50% reservoir solution, or 50% ethylene glycol (w/v) and 50% reservoir solution. Crystals were soaked for 5–6 min before flash cooling in LN_2_. Crystals grew to approximately 200×150×100 μm^3^ in space group C222_1_ with unit cell parameters a = 99.16, b = 217.37, c = 125.02 Å and α = β = γ = 90°. There are four molecules per asymmetric unit with an estimated solvent content of ∼ 58%.

Tetragonal crystals were obtained as above, with ∼2 mM GTP substituted for ATP in the hanging drop. Crystals grew to approximately 200×200×100 μm^3^ in space group P4_1_, with unit cell parameters a = b = 111.25, c = 129.12 Å and α = β = γ = 90°. There are four monomers per asymmetric unit with an estimated solvent content of ∼66%.

## Structure Determination and Refinement

### BtTLP/ATP cocrystal

A complete 2.35 Å data set was collected on a single crystal of BtTLP bound to ATP at 100 K at a wavelength of 1.03Å on a MAR m-300 CCD at beam-line 23 ID-B at the Advanced Photon Source (APS). Data were processed using HKL2000 [20]. The initial structure was solved by MOLREP in CCP4 [21], using the unliganded hTHG1 dimer (PDB code 3OTD) devoid of all non-protein atoms as the starting model. Rigid body refinement and further rounds of refinement were performed with Crystallography and NMR System (CNS) 1.2 [22]. Each round of refinement included energy minimization and B-factor refinement. Manual building was then performed in COOT [23]. Water molecules were then added and evaluated in COOT. Final stages of refinement were performed using Phenix with structure quality gauged using the MolProbity plugin within Phenix.refine [21], [24], [25]. Data processing and refinement statistics are shown in Table 1. The occupancy of the bound ATP varies slightly among the four monomers in the crystal asymmetric unit. In the Results and Discussion section the ATP-BtTLP interactions are described in detail for monomer C, as its bound nucleotide displays the highest occupancy.

**Table 1:** Crystallographic data collection and refinement statistics.

	ATP complex	GTP complex
PDB ID code	4KGM	4KGK
X-ray source	23-ID-B APS synchrotron	23-ID-D APS synchrotron
Space group	C222_1_	P4_1_
Cell dimensions		
a, b, c (Å)	99.16, 217.96, 126.06	111.25, 111.25 129.12
α, β, γ (°)	90, 90, 90	90, 90, 90
Resolution (Å)	50.0–2.35 (2.45−2.35)	40.0–2.96 (3.07–2.96)
Unique reflections	56,361	30,738
Redundancy a	6.0 (4.8)	2.8 (2.4)
Completeness a (%)	100 (99.7)	92.7 (80.2)
R_merge_ a (%)	8.2 (61.1)	9.6 (63)
I/σ a	21.1 (2.10)	8.5 (1.7)
**Refinement:**		
R_work/_R_free_^b^ (%) Twin law (fraction)	19.8/23.3–	22.9/25.8 (20.8/22.9)^c^ h,-k,-l (0.2)
r.m.s.d. bonds (Å)	0.003	0.002
r.m.s.d. angles (°)	0.71	0.64
Ramachandran (favored) (outlier)	97% 0%	96% 0%
Coordinate error (Å) maximum-likelihood	0.26	0.40
Average B-factors (Å^2^)	31.8	48.0 (62.2)^c^

a Values for the highest resolution shell are shown in parentheses. ^b^ *R*_free_ was calculated with 10% of the reflections not used in refinement. ^c^Refinement using twin fraction.

### BtTLP/GTP cocrystal

A 2.96 Å data set was collected at λ = 1.03 Å on a single crystal of BtTLP bound to GTP at 100 K and at 1.03 Å on a MAR m-300 CCD (MARResearch) at beamline 23 ID-D at the Advanced Photon Source (APS). Data were processed using HKL2000 [20]. The initial structure was solved by Phaser [26] in CCP4 [21], using the BtTLP/ATP dimer devoid of all non-protein atoms as the starting model. Intensity statistics indicated the presence of twinning. A twin law of h,-k,-l was identified and evaluated by Phenix.xtriage, and verified with the UCLA twinning server [27], [28] with a twin fraction later refined to 0.2. Free-R flags were selected using Phenix to ensure twin-related reflections were in the same group. The model could be suitably refined without inclusion of the twin fraction, while a modest improvement in R-factors and increase in B-factors was observed when included within refinement. Data processing and refinement statistics are shown in Table 1.

In the tetragonal crystals, the four monomers in the asymmetric unit represent the biological tetramer. In the orthorhombic crystals, the asymmetric unit is composed of two dimers that form two separate biological tetramers across their respective two-fold symmetry axes. RMSD calculations between subunits and/or different structures were performed using the Superpose program [29] in CCP4. All structure figures were made with PyMOL (The PyMOL Molecular Graphics System, Version 1.2r3pre, Schrödinger, LLC).

### Protein Data Bank Accession Codes

Atomic coordinates and structure factor amplitudes have been deposited with the Protein Data Bank (http://www.pdb.org) and are accessible under accession codes 4KGK (GTP complex) and 4KGM (ATP complex).

### *In vitro* activity assays

Single-turnover kinetic assays for determination of the rate of tRNA 5′-end activation were performed using *in vitro* transcribed tRNA substrates (full-length tRNA^His^ and either of two 5′-truncated tRNAs: tRNA^His^ ΔG_−1_/ΔU_+1_ or tRNA^Ile^ΔG_+1_, as indicated in each assay). tRNAs were 5′-labeled with ^32^P using T4 Polynucleotide kinase (NEB) and [γ-^32^P]-ATP as previously described [12]. Activation assays were conducted at pH 6.0 in a reaction buffer containing 25 mM NaOAc, 50 mM Bis-tris and 50 mM Tris-HCl pH 6.0, 10 mM MgCl_2_, 3 mM DTT, 125 mM NaCl and 0.2 mg/ml BSA.

The rate for activation of the 5′-end of tRNA by BtTLP was obtained using a previously described transient kinetic approach [3] by reacting ≤30 nmol of 5′-monophosphorylated [^32^P]-tRNA (p*tRNA; 6000 Ci/mmol) under single-turnover conditions with excess BtTLP enzyme (≥15 µM). To determine the k_obs_, the reactions were initiated by adding BtTLP and nucleotide (ATP or GTP, 0.1–1 mM, as indicated for each assay) to a preincubated mix of p*tRNA substrate in reaction buffer. The rate of product formation was measured by withdrawing aliquots (3 µl) from the reaction mixture, which were quenched using 80 mM EDTA and 1.5 mg/ml RNase A (Ambion) followed by 10–20 min incubation at 50°C. These aliquots were further treated with 0.5 U Calf intestinal phosphatase (Invitrogen) at 37°C. To resolve the reaction products, App*GpC (ATP-dependent activation) or Gpp*GpC (GTP-dependent activation), from the inorganic phosphate (P_i_*) derived from unreacted substrate, 2 µl of each quenched reaction was spotted on a silica TLC plate (EM Science) and the reaction products were separated using a 1-propanol:NH_4_OH:H_2_O (55∶35∶10, v/v/v) solvent system. The TLC plates were visualized using a Typhoon Trio phosphorimager and quantified using ImageQuant software (GE Healthcare) as described previously [12].

## Results and Discussion

### Structural Overview

**Figure 1:**
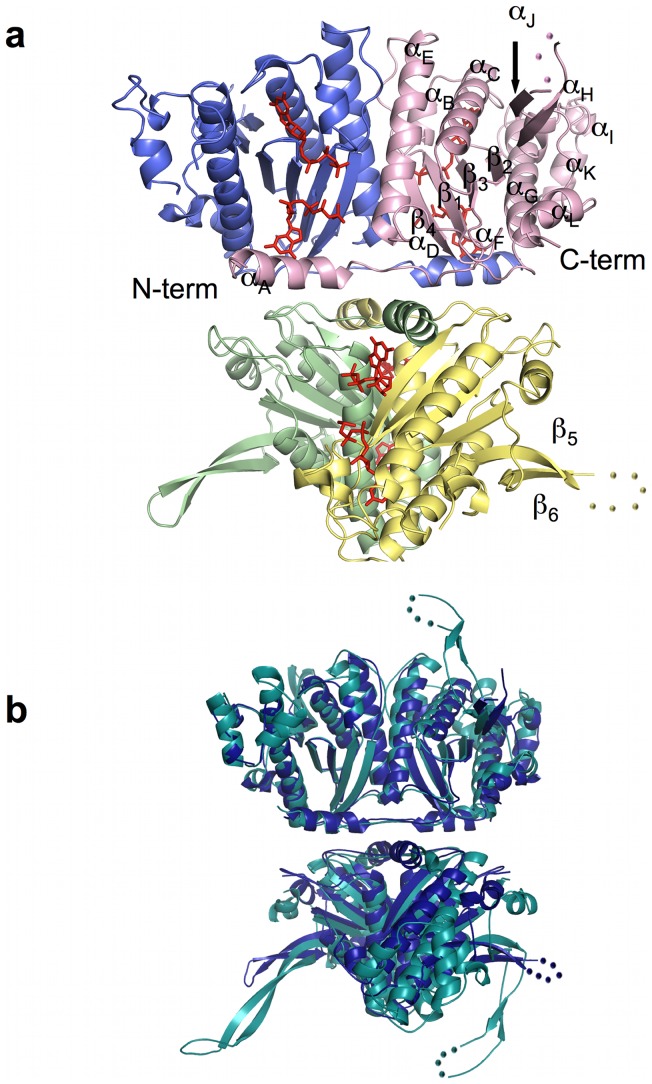
Ribbon diagram of the *Bacillus thuringiensis* tRNA^His^ guanylyltransferase (Thg1)-like protein (TLP) homotetramer. (a) The BtTLP tetramer consists of a dimer of dimers. Monomers are colored as follows: blue, monomer A; pink, monomer B; green, monomer C; and yellow, monomer D. Disordered residues are represented by spheres. GTP molecules are shown in red. (b) Superposition of BtTLP and human THG1. The overlay of BtTLP (dark blue) and hTHG1 (cyan) illustrates that the two enzymes adopt very similar quaternary structures.

A BtTLP monomer is composed of 245 amino acids, with an estimated molecular weight of 28.5 kDa. Similar to yeast and human Thg1, BtTLP is a tetramer consisting of a dimer of dimers (Figure 1A). Each monomer of BtTLP is broadly similar to that of hTHG1, with an RMSD of 1.45 Å [29]. The tetrameric organization is also very similar to that described for hTHG1 (Figure 1B) [16]. Crystal structures of the bacterial enzyme were obtained in the presence of either ATP or GTP. The structure of BtTLP bound to ATP was solved to a resolution of 2.35 Å in an orthorhombic space group, and to 2.96 Å in tetragonal crystals grown in the presence of GTP (Table 1). The initial ATP-bound BtTLP structure was solved via molecular replacement, using the hTHG1 dimer as a starting model [16]. The complex with GTP was solved by molecular replacement using the ATP-bound model.

**Figure 2:**
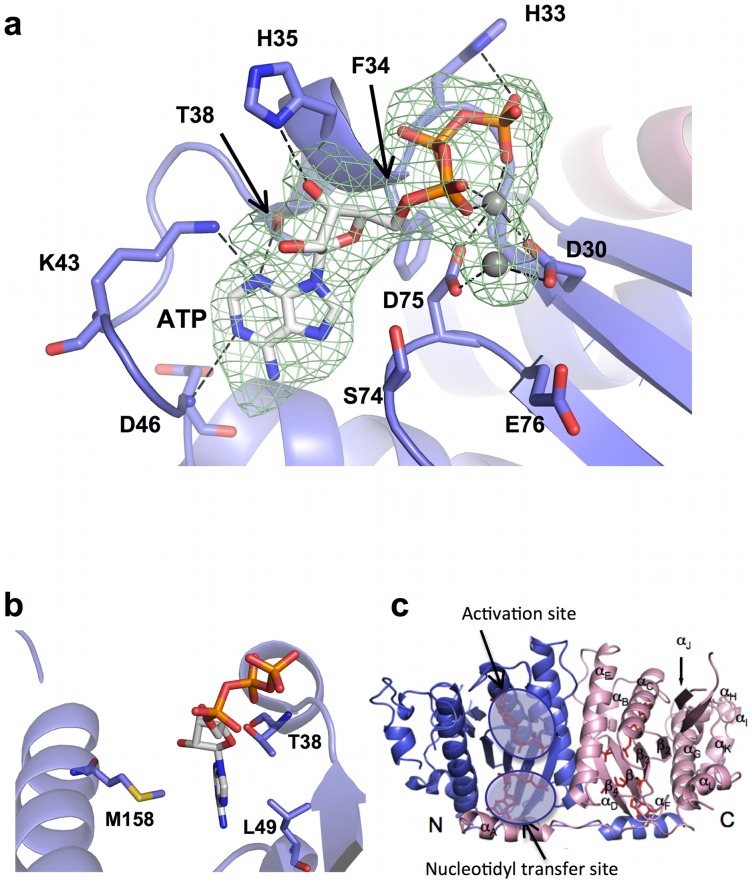
ATP is bound in the activation site. (a) Close-up view of ATP bound in activation site. Several catalytically important residues contact the nucleotide. The triphosphate tail ligates two metal ions (shown as gray spheres). A Fo-Fc omit map (green mesh) is contoured at 3 σ. (b) View rotated ∼90° compared to (a). The purine base is sandwiched between M158 on one side and T38 and L49 on the other side. (c) The inset indicates the location of the activation and nucleotidyl transfer sites in the GTP-bound model. The ATP nucleotide is only observed in the activation site.

Sequence alignments with human and yeast Thg1 proteins show that BtTLP shares significant sequence similarity to hTHG1 and ScThg1 (overall identity between TLP and Thg1 sequences is about 40%), including many of the residues known to be essential for enzymatic activity of the eukaryotic enzymes [3], [10], [30] (Figure S1). For example, the three conserved carboxylates observed in the BtTLP active site (D30, D75 and E76) correspond to D29, D76, and E77 in hTHG1 [16]. As with the human enzyme, D30 and D75 coordinate two Mg^2+^ ions, which in turn contact the triphosphate tail of a bound nucleotide (Figure 2). Consistent with human and ScThg1, alteration of the metal-coordinating D75 in BtTLP to alanine results in dramatically decreased enzymatic activity [16], [30] (Figure S2). Thus, BtTLP likely uses a two-metal ion mechanism for the various steps of the 3′–5′ addition reaction, similar to the mechanism predicted for eukaryotic Thg1 enzymes.

In addition to the metal-coordinating carboxylates, previous structural and kinetic investigation of human and ScThg1 identified distinct residues that play important roles in either the first (5′-end activation) step of the reaction (K44, S75 and N161 of ScThg1; which are K43, S74, and N158 in hTHG1) or the second (nucleotidyl transfer) step of the reaction (R27, K96 and R133 of ScThg1; which are R27, K95 and R130 in hTHG1) (Figure S1) [3], [16]. The differing effects of alterations of these residues on separate steps of the Thg1 reaction led to the proposal that these residues define at least partially separable active sites for the activation and nucleotidyl transfer steps, respectively. Accordingly, a completely visualized dGTP molecule in the published hTHG1 structure (PDB ID code 3OTB; [16]) reflects the position of the activating NTP used for the first step of catalysis, while a second, partially visualized, dGTP reflects the position of the incoming NTP used for the nucleotidyl transfer step of the reaction. Identical amino acids to those in ScThg1 are found at five out of these six positions of BtTLP (all but N161) (Figure S1), suggesting overall similarity in the mechanism of these two steps between the two families of enzymes. However, the structural data described below reveal additional interactions that appear to be important for the function of Thg1 family enzymes. For consistency, unless otherwise indicated, amino acid numbering used throughout the remainder of this paper refers to the amino acid position of the residues in BtTLP, with the corresponding residues in hTHG1 or ScThg1 indicated in parentheses, as needed.

### BtTLP exhibits nucleotide and tRNA-specific kinetic differences in the activation step compared to eukaryotic Thg1

Previous structural and biochemical data provided strong support for the involvement of one of the hTHG1-bound nucleotides in the activation step of the reaction. Yet, differences observed between the 5′-end activation reaction catalyzed by BtTLP compared with activation catalyzed by yeast Thg1 suggested that a more detailed investigation of this reaction would be necessary to understand the nucleotide-bound BtTLP structures [12], [31]. In the earlier experiments, BtTLP used either ATP or GTP for activation of a tRNA 5′-end. These data contrast with the very low levels of 5′-end activation with GTP observed with eukaryotic Thg1 enzymes, and thus imply additional flexibility in the BtTLP active site relative to its eukaryotic counterparts [2]. Pyrimidine nucleotides are not used to activate the 5′-end of any substrate to significant levels by either enzyme.

**Figure 3:**
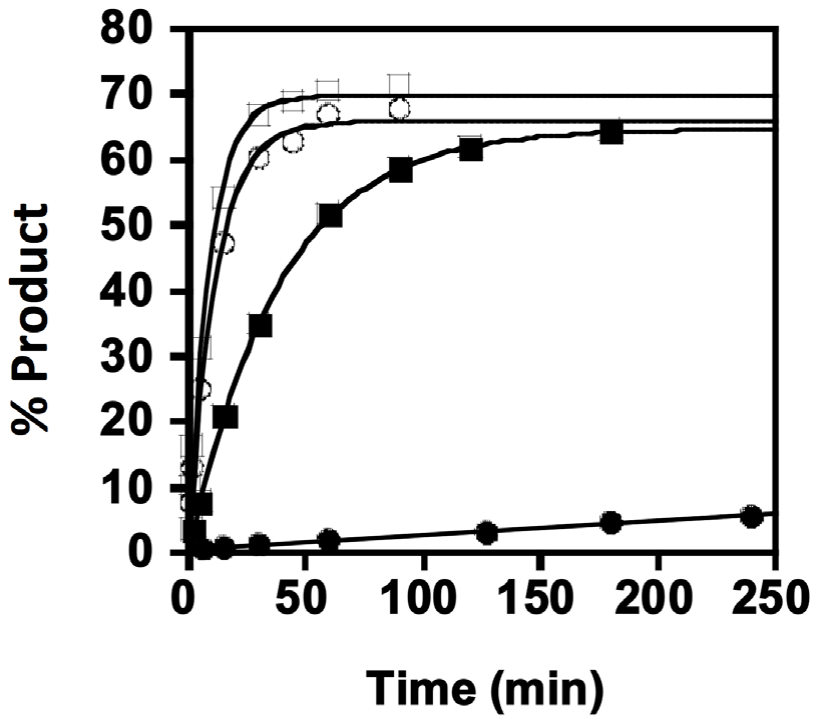
Kinetics of BtTLP-catalyzed 5′-end activation. Single-turnover measurements for activation of either 5′-truncated (open symbols) or full-length (closed symbols) 5′-^32^P labeled tRNA^His^ substrates were determined with 15 µM BtTLP in the presence of either 1 mM ATP (○ or •) or 1 mM GTP (□ or ▪) to yield k_obs_ for each condition. Fits shown are to the single-exponential rate equation for all data except for ATP-dependent activation of full-length tRNA, for which the method of linear initial rates was used to determine k_obs_.

Single-turnover kinetic measurements allow quantification of microscopic rate constants for individual chemical steps on an overall reaction pathway by performing kinetic assays using a large excess of enzyme over substrate, and thus limiting the observed reactions to a single reaction of each enzyme-bound species. Thus, to quantify the relative use of ATP vs. GTP nucleotides for 5′-end activation by BtTLP, we adapted a single-turnover kinetic assay previously developed to isolate the adenylylation step of the reaction [3], and directly compared the kinetics of the adenylylation and guanylylation steps catalyzed by BtTLP. Observed rate constants (k_obs_) were measured with two different 5′-[^32^P]-labeled tRNAs using high concentrations of enzyme (15 µM) at pH 6.0, which is the optimal pH for ATP-dependent activation catalyzed by ScThg1. Interestingly, when full-length tRNA^His^ (lacking the G_−1_ residue) was used as a substrate, BtTLP exhibited a strong preference for use of GTP over ATP for 5′-end activation (Figure 3, filled symbols) [2]. While the rate of guanylylation was readily determined from the single-exponential fit to the data (k_obs_  = 0.026±0.0003 min^−1^), product formation was extremely slow in the presence of only ATP, and the method of linear initial rates was used to estimate k_obs_  = ∼0.0003 min^−1^.

Activation rates were then measured using a second tRNA, a 5′-truncated substrate missing the +1 nucleotide, which is one of the kinetically-preferred substrates for BtTLP activity [12]. Using the 5′-truncated tRNA, the k_obs_ for 5′-end activation by GTP was only slightly increased (0.11±0.01 min^−1^) relative to k_obs_ for GTP-activation of the full-length tRNA (Figure 3, compare open vs. filled squares). However, the k_obs_ for activation of the 5′-truncated tRNA by ATP was considerably enhanced over the rate of the reaction with full-length tRNA, to 0.089±0.006 min^−1^ (Figure 3, compare open vs. filled circles). Thus both ATP and GTP are used with relatively equal efficiency for activation of a repair-type substrate by BtTLP. These data reveal yet another biochemical difference between TLPs and eukaryotic Thg1-type enzymes, since the ability to efficiently use GTP for the first (activation) step of the reaction appears to be a unique property of TLP members of the enzyme family.

In light of this demonstrated flexibility with respect to purine nucleotide use, it is significant that the crystal structures of BtTLP reported here have been obtained in separate complexes with ATP and GTP, as opposed to just the guanosine nucleotide-bound complex that was obtained with hTHG1. In the analysis of the two nucleotide-bound forms of BtTLP described below, we aimed to identify features that distinguish the mode of binding of the two purine nucleotides in the activation site in order to understand the distinct ways in which these two nucleotides are used in this step of catalysis. In doing so, we emphasize that these structures of BtTLP add additional structural evidence to support the identification of the conserved nucleotide binding pocket as the binding site for the activating nucleotide required to create a high-energy phosphoanhydride bond for 3′–5′ addition to 5′-monophosphorylated substrates.

### Insights from the GTP-bound complex: Complete GTP nucleotides in the activation and nucleotidyl transfer sites

**Figure 4:**
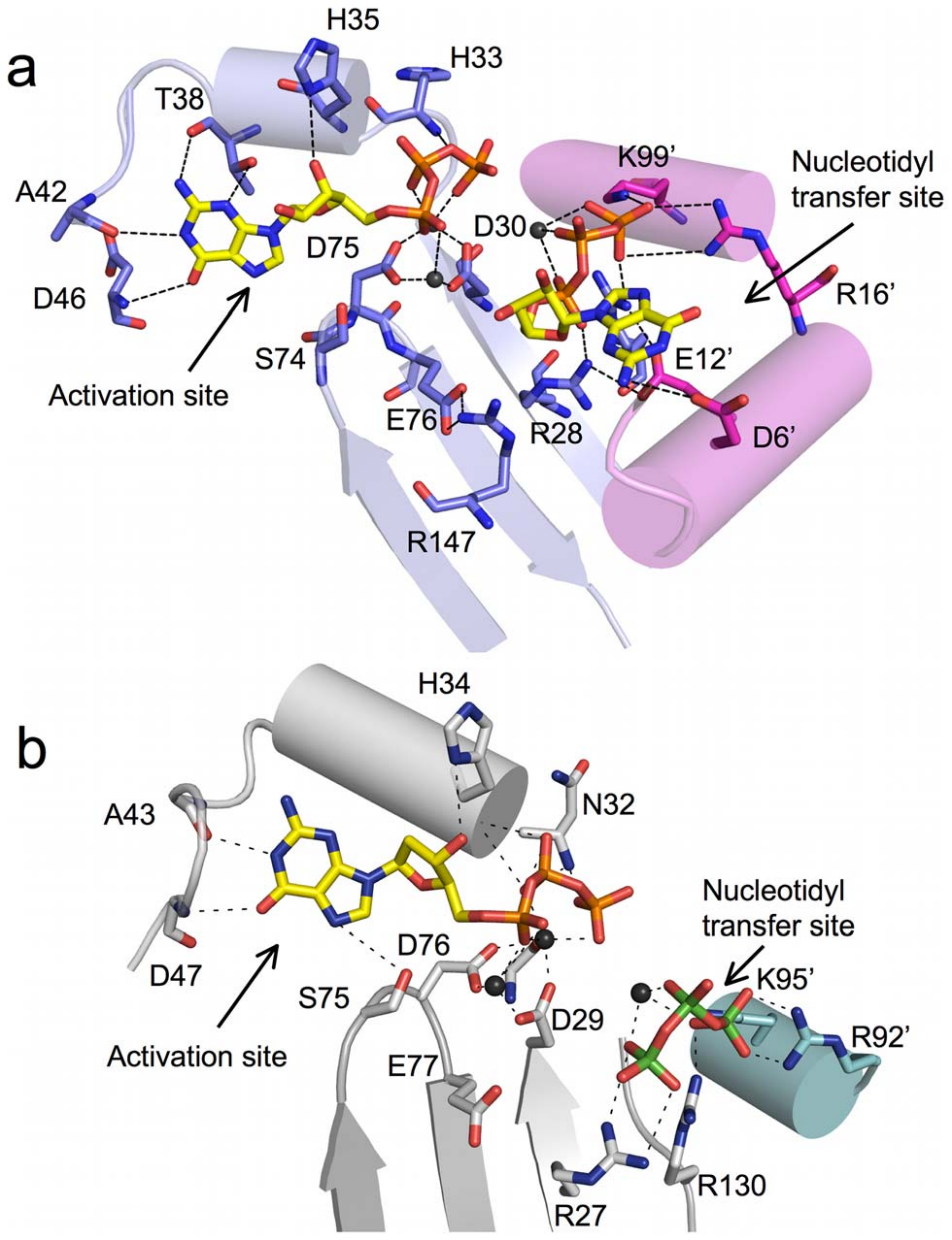
GTP molecule is bound in both activation and nucleotidyl transfer sites. (a) Two GTP molecules were observed in the BtTLP structure. The interactions with the guanine base in the activation site come mostly from protein main-chain atoms. Several conserved, catalytically important residues interact with the triphosphate tail in the nucleotidyl transfer site. Some of the interactions to the triphosphate and base originate from the adjacent monomer (Residues shown in pink). (b) Activation and nucleotidyl transfer sites of hTHG1. The two enzymes employ similar residues to bind GTP in both sites. Residues originating from the adjacent monomer are shown in teal.

As with hTHG1, a co-crystal structure of BtTLP containing two bound GTP nucleotides has been obtained. In the BtTLP/GTP complex a GTP molecule is observed in the activation site for all four monomers, bound in a similar orientation to the dGTP nucleotide in the hTHG1/dGTP complex. A second complete GTP is seen in two of the four subunits of the BtTLP tetramer (monomers A and B), with a second partial GTP observed for monomers C and D. (Figure 4A and Figure S3). In contrast, only the triphosphate portion of the second bound nucleotide was observed in the hTHG1 complex (Figure 4B).

In the activation site, the Watson-Crick face of the guanine interacts with several main chain atoms (Figure 4A). The amide of D46 participates in a hydrogen bond with O6 of guanine, and the carbonyl moiety of A42 interacts with N1, identical contacts to those observed in the hTHG1/GTP structure (Figure 4B). In the BtTLP/GTP structure, however, there are additional contacts between T38 and the bound GTP, with main chain carbonyl-N2 and OG1-N3 interactions provided by this residue. In hTHG1, the analogous residue is an alanine (A37) and therefore is not able to similarly interact with N3.

Two other interactions with the bound GTP, H35(34) with the 3′-OH and S74(75) with the GTP-N7, are also similar to interactions observed in the hTHG1/dGTP structure (Figure 4). Alteration of the analogous histidine residue (H34) in hTHG1 to alanine did not cause any discernible effects on activation or nucleotidyl transfer steps of the G_−1_ addition reaction, suggesting that this interaction with the ribose is not critical for catalysis [16]. The fact that the His35-3′-OH interaction is conserved between human and bacterial Thg1-family enzymes would not have been readily predicted from sequence alignments, since the histidine residue at this site is much more sporadically observed among bacterial and archaeal TLPs (Figure S1). It will be interesting to characterize additional TLP-NTP structures to see whether this interaction is maintained in other family members that contain a histidine nearby in the sequence, but not identifiably at this site. In contrast, the serine residue that contacts the nucleotide (S74 in hTHG1) is important specifically for the adenylylation step catalyzed by Thg1, and the conserved nature of the S75-GTP interaction further supports the assignment of this nucleotide as representing the position of the activating NTP in catalysis [3]. Taken together, the many similarities between the activation site of BtTLP and hTHG1 suggest that these enzymes use similar mechanisms for the activation step of the reaction. The basis for the ability of BtTLP to utilize GTP in addition to ATP for the activation step of the 3′–5′ addition reaction, as compared to the inability of hTHG1 to efficiently do so, is not entirely clear from the structural data. The additional contact observed between T38 and the GTP base, as compared to the more limited interactions possible with the analogous A37 residue in hTHG1, may provide some structural rationale for the observed difference. However, we note that several other eukaryotic Thg1 enzymes (including ScThg1, which does not efficiently use GTP for activation) similarly have a hydroxyl-containing residue (S or T) at this position (Figure S1).

In contrast to the hTHG1/dGTP structure where the second bound dGTP nucleotide was highly disordered so that only the 5′-triphosphate moiety was visible in the electron density map (Figure 4B), the GTP-bound BtTLP structure reveals complete density for the second nucleotide (Figure 4A). The triphosphate tail of this second GTP observed in BtTLP overlays with the triphosphate seen in hTHG1 (PDB code 30TB; [11], [16]). Previous kinetic data suggested that this second NTP-binding site is associated with the nucleotidyl transfer step (step 2) of the 3′–5′ addition reaction [3]. As with the activating GTP described above, the interactions with the triphosphate moiety of the second GTP are similar for bacterial and human enzymes, involving multiple positively charged residues (Figures 4 and S4). The α-phosphate forms a salt bridge with R28 and K99′ (of the adjacent monomer in the dimer). The γ-phosphate participates in salt bridges with three residues: R131, R16′, and K99′. By analogy to hTHG1, we propose that this second bound NTP represents the position of the incoming NTP to be added to the polynucleotide chain, which is supported experimentally by dramatic decreases in the kinetic efficiency of the nucleotidyl transfer step after alteration to alanine of several of the analogous positively charged residues in yeast Thg1 (R27, K96 and R133, which correspond to R28, K99 and R131 in BtTLP) [3].

Despite the ability to observe the complete bound GTP nucleotide in the BtTLP structure, we detected no direct contacts to the ribose moiety and few to the base itself that suggest a mechanism of selection of the incoming NTP during nucleotidyl transfer (Figures 4 and S4). N2 of guanine hydrogens bond with D6 of the adjacent BtTLP molecule; yet, this residue is not highly conserved (Figure S1) and most Thg1/TLP enzymes lack negatively charged residues at this position. Although the effects of altering D6 in BtTLP have not been measured, and thus the possibility for a unique role for this interaction can not be excluded, these data suggest that the orientation of the nucleotide base of the presumed incoming NTP observed in the nucleotide transfer site likely does not reflect the position of the base during 3′–5′ addition. In the absence of a tRNA substrate to provide a templating strand (for either G_−1_•A_73_ or G_−1_•C_73_ base pairing), interactions with protein residues may replace base pairing interactions between the nucleotide bases. A structure obtained in the presence of tRNA is likely required for visualizing the active conformation of the nucleotide base in the nucleotidyl transfer site. The structural data do, however, support the role of interactions with the triphosphate tail in keeping the GTP in place for addition of the nucleotide to the adenylylated tRNA intermediate.

The presence of a second complete nucleotide bound to BtTLP does, however, elucidate the previously unexplained role of a number of additional residues shown to be critical in ScThg1 or hTHG1 mutational studies [10], [16], [30]. In family A polymerases, such as T7 DNA polymerase, two of the three catalytic carboxylates coordinate Mg^2+^ ions, which interact directly with the triphosphate tail of the incoming nucleotide [32]. The hTHG1 structure showed the same arrangement in the putative activation site [16]. The function of the third carboxylate in hTHG1 (E77) remained unclear, as it is pointing away from the nucleotide and does not interact with either Mg^2+^ ion. In the BtTLP structure, however, the side chain of the third carboxylate, E76, is located roughly 4 Å away from the aliphatic chain of R28, a residue shown to be important in binding the incoming GTP molecule [3] (Figure 4A). The van der Waals interaction with E76 may stabilize the side chain of the arginine residue, without interfering with its ability to hydrogen bond with other residues, and anchoring it in a way that allows NH1-2 to interact with the α-phosphate of the second GTP molecule. E76 also participates in a salt bridge interaction with R147, a residue whose guanidinium group stacks with that of R28 (Figure S4). Arginine 147 is strictly conserved and the ScThg1 R147A variant has 0.9% of the wild-type enzyme activity [30], although the specific function of R147 in catalysis has not been fully investigated.

The charged and highly conserved residue E12 (E13 in ScThg1) was also shown by mutational studies to be important for enzymatic activity in yeast Thg1 [30]. It was not readily apparent from the hTHG1 structure why this residue is important for catalysis. In the BtTLP structure, however, the carboxylate group of E12 participates in salt bridge interactions with two arginines, R28 and R131, both of which contact the triphosphate tail of the GTP in the nucleotide transfer site (Figure S4). Thus, alteration of E12 could indirectly affect positioning of the incoming NTP, and the effect of this alteration is expected to be primarily observed on the nucleotidyl transfer step of the reaction. Further kinetic analysis of this variant for specific defects in catalysis of 3′–5′ addition will be needed to verify this hypothesis.

### ATP molecule is visualized for the first time in the activation site

**Figure 5:**
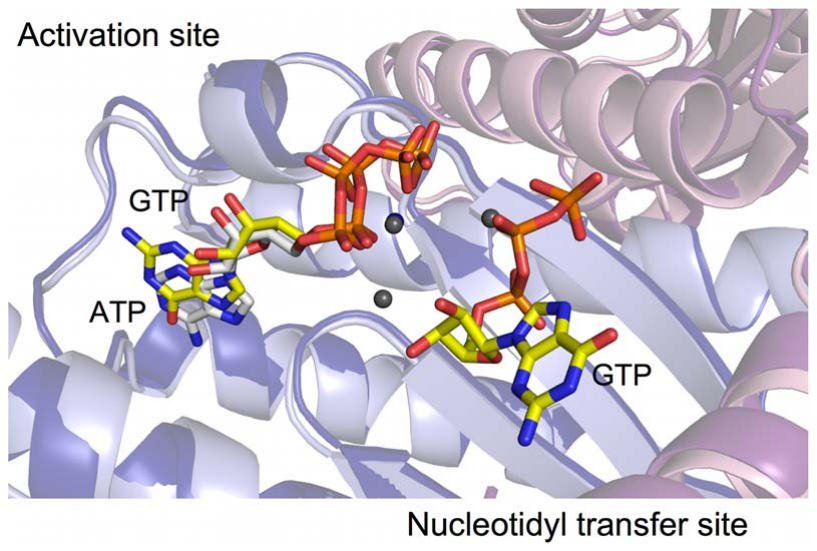
Superposition of the GTP- and ATP-bound models of BtTLP. The ATP complex (shown in light blue (monomer A) and pale pink (monomer B) with nucleotide in white) has one nucleotide bound in the activation site whereas the GTP complex (dark blue and pink (monomers A and B), with GTP in yellow) revealed two bound nucleotides, one in the activation site and the other in the nucleotidyl transfer site. The superposition between the two nucleotide-bound models revealed that although the triphosphate moieties and metal ions are almost indistinguishable there is a noticeable shift in the position of the purine base, presumably to accommodate different hydrogen-bonding partners.

Unlike for hTHG1, which did not yield diffraction-quality co-crystals in the presence of any ATP nucleotide or derivative, BtTLP yielded 2.4 Å diffracting crystals in the presence of ATP. Importantly, one ATP molecule is bound in the site implicated in the activation step (Figure 2C) by the previous kinetic and structural characterization of human and ScThg1, in similar orientation to the bound GTP visualized in this site in the other structures (Figure 5). As with the structures that contain GTP at this site, the triphosphate tail ligates the two metal ions, which in turn contact the two strictly conserved aspartate residues, D30 and D75 (Figure 2). Two non-bridging oxygens (phosphates β and γ) hydrogen bond with two main-chain amides (H33 and F34). As with the GTP-bound structure discussed above, the 3′-OH of the ribose moiety hydrogen bonds with His35 and there is no direct contact to the 2′-OH, again consistent with a lack of role for the 2′-OH of the activating nucleotide in the chemistry of the adenylylation reaction [2]. Thus, the interactions between BtTLP and the sugar and triphosphate moieties remain largely the same in the ATP- and GTP-bound structures (Figure 5).

The interaction between BtTLP and the adenine base reveals both similarities and differences to the aforementioned GTP-bound structures. Consistent with the previous structural and kinetic data, the close contact between S74 and ATP-N7 is also evident in the ATP-bound structure (Figure 2). Also, the contact between N3 and the side chain hydroxyl of T38 is maintained in the ATP-bound structure. A superposition of the two bound nucleotide bases reveals a displacement of 1 to 1.5 Å between the adenine and guanine base (Figure 5). Because of this shift, the amide group of D46 is now within hydrogen bonding distance of the ATP-N1, instead of the C6 carbonyl of GTP (Figure 2). Thus, as predicted from the hTHG1 structure, main-chain interactions with the base afford BtTLP the flexibility to interact with either purine base (Compare Figure 2 and Figure 4A).

Most importantly, the ATP-bound structure reveals a direct hydrogen bonding interaction between N3 of the bound ATP and the side chain of a highly conserved lysine, K43 (Figure 2). The amino group of the analogous hTHG1 residue (K44) was located far away from the guanine base (about 7 Å) in the hTHG1 crystal structure, yet this conserved lysine was implicated kinetically in the activation step catalyzed by ScThg1 [3]. Therefore, these new structural data rationalize kinetic involvement of K43 (K44) in the 5′-end activation of the tRNA by suggesting an active role for this residue in positioning the activating ATP. Interestingly, K43 does not interact with the guanine base in the BtTLP GTP-bound structure, as there would likely be a steric clash between the lysine side chain and the exocyclic amine of the base. One prediction from these results is that the use of GTP for activation by BtTLP would be independent of the presence of the K43 side chain. Kinetic analysis of the K43A variant revealed that indeed, the observed rate of adenylylation, measured with the 5′-end repair substrate so that quantifiable reaction rates are observed (Figure 3), was significantly decreased (by ∼10-fold), while the rate of guanylylation was virtually unchanged as a consequence of the K43A alteration (Table 2). Thus, the current structure advances our understanding of the activation step by providing direct evidence for contacts between the purine ring of the bound ATP and this lysine residue. This interaction, combined with the significant defects in the K_D,app_ for the activating ATP nucleotide with the K44A ScThg1 variant (but notably not the K_D,app_ for the incoming GTP nucleotide for G_−1_ addition) underscores the important role for K43/K44 in binding and/or positioning the ATP for the activation step.

**Table 2:** Kinetics of 5′-end activation catalyzed by BtTLP variants.

BtTLP	tRNA substrate	activating NTP	k_obs_ (min^−1^)
wild-type	full-length^a^	GTP (1 mM)	0.046±0.007
M158A	full-length	GTP (1 mM)	0.17±0.01
M158N	full-length	GTP (1 mM)	0.44±0.03
wild-type	full-length	ATP (2 mM)	∼0.0007*^c^*
M158N	full-length	ATP (2 mM)	∼0.001*^c^*
wild-type	ΔG_+1_ *^b^*	ATP (0.3 mM)	0.91±0.14
K43A	ΔG_+1_	ATP (0.3 mM)	0.12±0.01
wild-type	full-length	GTP (0.1 mM)	0.022±0.004
K43A	full-length	GTP (0.1 mM)	0.022±0.008

a Substrate is full-length *S. cerevisiae* tRNA^His^ (missing G_−1_ residue) used for standard G_−1_ addition assays [30]. *^b^* Substrate is *D. discoideum* mitochondrially-encoded tRNA^IleCAU^ missing G_+1_ residue (transcript initiates with C_+2_) used for standard tRNA repair assays [13]. *^c^* Estimate for k_obs_ values derived from observed rates determined using the method of linear initial rates.

Unlike the complex with GTP, which revealed two GTP molecules bound, only the activation site has a full nucleotide in the ATP-bound complex. A phosphate moiety was modeled in the nucleotidyl transfer site in each of the 4 monomers of BtTLP. The phosphate is in contact with Arg131, Arg16′, and Lys99′ of the adjacent monomer. This corresponds to the position of the γ-phosphate of the full nucleotide bound in the GTP complex. The fact that we observe two nucleotides bound in the BtTLP GTP complex vs. only one complete nucleotide in the ATP complex could be due to differences in the space groups and crystal packing environment, as well as in the crystallization buffer compositions (2 mM GTP vs. 1 mM ATP).

### Investigation of the use of both GTP and ATP nucleotides for 5′-end activation by BtTLP

In the structure of BtTLP bound to ATP, the adenine base is sandwiched between M158 on one side and T38 and L49 on the other (Figure 2B). Similar interactions are seen with the guanine base in the GTP-bound structure (not shown). In the hTHG1 structure, however, the guanine base only has van der Waals interactions on one side (F42 of hTHG1 occupies about the same position as L49 of BtTLP), and the analogous residue to M158 is the small polar residue N158. Moreover, N161, the analogous residue at this position in ScThg1, plays a critical role in adenylylation, but not other steps of the ScThg1-catalyzed G_−1_ addition reaction [3]. We therefore tested whether the M158 residue is important for activation by altering this residue to either alanine, or to the eukaryotic asparagine. Interestingly, removal of the M158 side chain (M158A alteration) had no effect on the observed rate of activation; in fact, the k_obs_ is slightly enhanced (Table 2). Thus the highly conserved N161 (N158) residue in yeast and human Thg1 appears to play a role in a eukaryote-specific aspect of the activation step. We also tested whether introduction of the eukaryotic asparagine in place of M158 caused BtTLP to adopt the eukaryote-like pattern of activation (preference for ATP over GTP for the activation step). In fact, the BtTLP M158N variant was not only unable to impart the eukaryotic preference for ATP to the enzyme, but it further improved the kinetics for GTP-dependent activation by 10-fold relative to the wild-type BtTLP (Table 2). These results suggest that M158 in the BtTLP enzyme is not a controlling factor for the ability of BtTLP to use GTP preferentially over ATP for activation.

Taken together, of the three yeast Thg1 residues K44, S76, and N161 (corresponding to K43, S74 and M158 in BtTLP) shown to be important for the initial activation step of the reaction catalyzed by ScThg1 (Figure S1), we have provided new structural data to rationalize a direct role for K44 in catalysis (based on the ATP-bound BtTLP structure) and reaffirmed the important role of S76 based on the conserved nature of the interactions between the serine residue and N7 of the activating NTP in both ATP- and GTP-bound structures obtained here. These roles appear likely to be universal functions for these residues among all members of the Thg1/TLP enzyme family. In contrast, the role for the third residue implicated to function in adenylylation is likely to be eukaryotic Thg1-specific, since the residue located at the analogous position in BtTLP (M158) is not essential for the activation reaction catalyzed by BtTLP.

## Conclusions

We report the first crystal structure of a Thg1-like protein (TLP) that catalyzes a distinct biological function apart from tRNA^His^ maturation. In contrast to eukaryotic Thg1 enzymes, TLP enzymes prefer to catalyze repair of tRNAs with damaged 5′-ends, making them well-suited to participating in tRNA quality control pathways *in vivo*. Consistent with the shared ability of both Thg1 and TLP enzymes to catalyze 3′–5′ polymerase activity, the overall fold of BtTLP is very similar to that of human THG1. The overall conserved architecture suggests that the similarity to canonical polymerases is a property of the earliest ancestors of this enzyme family, and is unrelated to tRNA^His^ metabolism. In this work, we captured structures of BtTLP bound to two of its substrates, ATP and GTP. The ATP-bound structure provides insight into the role of a highly conserved lysine residue (K43 in BtTLP) in binding to the ATP nucleotide that is used for the adenylylation reaction catalyzed by Thg1/TLP enzymes. As suggested by this new structural data, the role of K43 is unique to the adenylylation reaction, and use of GTP for the activation step does not depend on the presence of this residue. We also observed for the first time a complete nucleotide bound in the nucleotide transfer site. Further insight into what makes an enzyme prefer to catalyze repair (TLP) vs. G_−1_ addition (Thg1) will require future studies identifying interactions with a tRNA substrate.

## Supporting Information

Figure S1 **Sequence alignment of Thg1 (top four species) and TLP (bottom six species) enzymes.** Strictly conserved residues are shown on a black background. Organisms used for the alignment (with accession numbers for each protein sequence shown in parentheses) are: S. cer., *Saccharomyces cerevisiae* (NP_011538.1); H. sap., *Homo sapiens* (NP_060342.2); D.mel, *Drosophila melanogaster* (NP_609737.1); D. dis. *Dictyostelium discoideum* (XP_629958.1); M. bar., *Methanosarcina barkeri* (YP_305268); M. the., *Methanobacterium thermoautotrophicum* (NP_276107); H. but., *Hyperthermus butylicus* (YP_001013237); S. cel, *Sorangeum cellosum* (YP_001616706); M. xan., *Myxococcus xanthus* (YP_634103); B. thu., *Bacillus thuringiensis* (ZP_00738534.1). The number of C-terminal amino acids omitted from each protein sequence is indicated in parentheses at the end of each line in the alignment. The three conserved carboxylates are shown in black, the residues interacting with the triphosphate tail of the nucleotide bound in the nucleotidyl transfer site are shown in blue. The residues involved in the activation step are shown in red. Residues in yellow are conserved amino acids whose function was explained by the current BtTLP structures.
(TIFF)

Figure S2 **Mutating the metal-coordinating D75 in BtTLP to alanine results in a dramatically decreased enzymatic activity.** Phosphatase protection assay of purified *Bacillus thuringiensis* TLP (BtTLP) (wild-type and D75A variant) for G_−1_ addition activity with 5′-^32^P-labeled tRNA^His^. Addition of G_−1_/additional G-nucleotides results in production of phosphatase-resistant oligonucleotide products, as indicated to the right of the figure; in the absence of 3′-5′ addition activity, the labeled phosphate is removed by phosphatase treatment after the reaction and visualized as inorganic phosphate (P_i_). Assays contained 5-fold dilutions of each purified enzyme (∼1–0.008 mg/ml). Lane Bt, control G_−1_ addition reaction with previously-purified BtTLP; lane Sc, control G_−1_ addition reaction with *Saccharomyces cerevisiae* Thg1 (ScThg1); lane -, buffer control.
(TIF)

Figure S3 **Close-up view of the activation and nucleotidyl transfer sites in the GTP-bound BtTLP model with overlaid Fo-Fc map (green mesh) contoured at 3 σ.**
(TIF)

Figure S4 **Close-up view of the BtTLP nucleotidyl transfer site and second GTP.** The view was rotated compared to Figure 4A in order to display all enzyme-nucleotide interactions. The triphosphate moiety of the GTP in the activation site is seen on the left.
(TIF)
