# Supplementary figures and images for: Structural Studies of a Bacterial tRNAHIS Guanylyltransferase (Thg1)-Like Protein, with Nucleotide in the Activation and Nucleotidyl Transfer Sites
Source: PLoS One. 2013 Jul 3;8(7):e67465. doi: 10.1371/journal.pone.0067465 (PMC3701042; doi:10.1371/journal.pone.0067465)

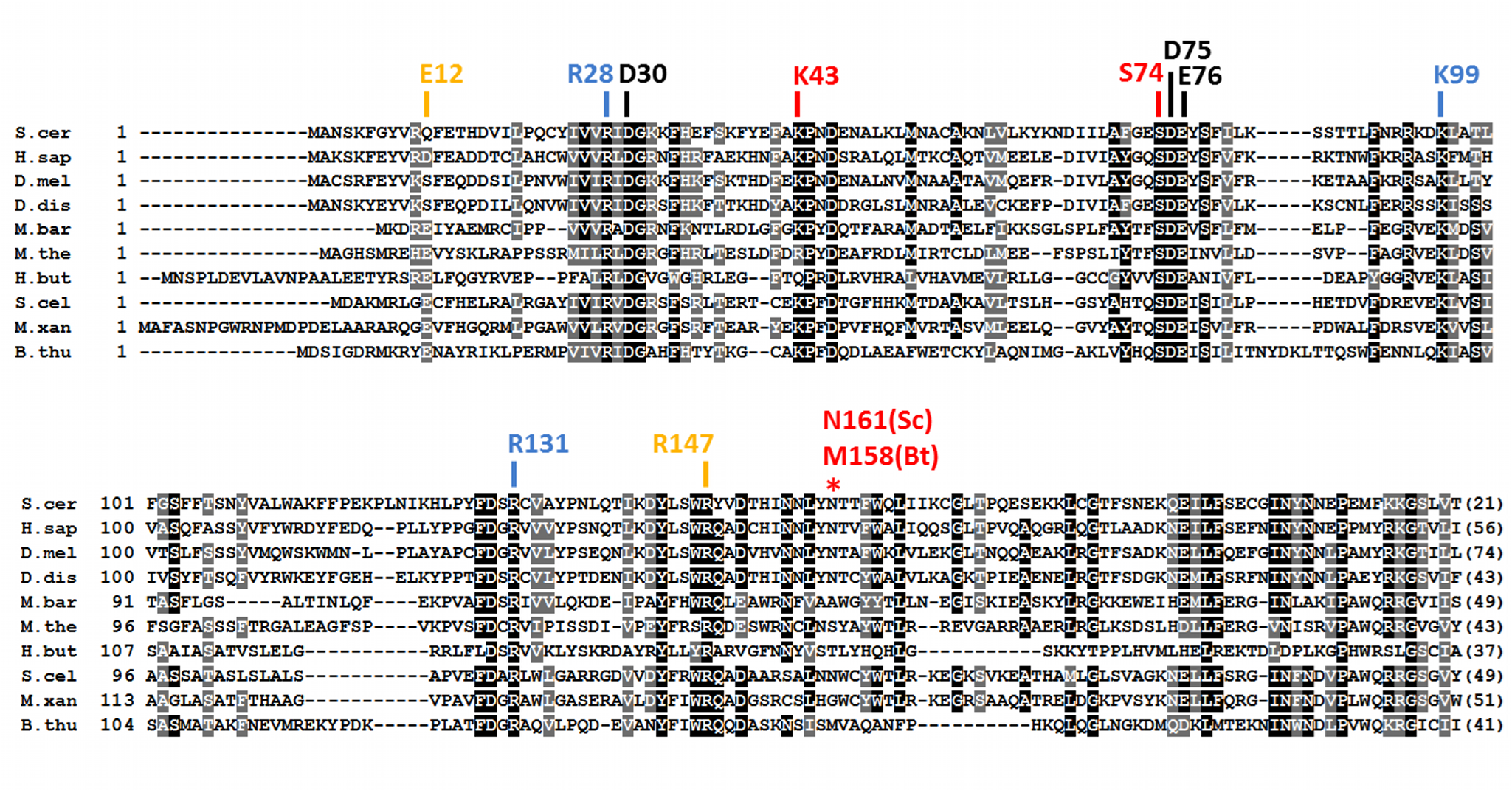

Supplement: Figure S1 — Sequence alignment of Thg1 (top four species) and TLP (bottom six species) enzymes. Strictly conserved residues are shown on a black background. Organisms used for the alignment (with accession numbers for each protein sequence shown in parentheses) are: S. cer., Saccharomyces cerevisiae (NP_011538.1); H. sap., Homo sapiens (NP_060342.2); D.mel, Drosophila melanogaster (NP_609737.1); D. dis. Dictyostelium discoideum (XP_629958.1); M. bar., Methanosarcina barkeri (YP_305268); M. the., Methanobacterium thermoautotrophicum (NP_276107); H. but., Hyperthermus butylicus (YP_001013237); S. cel, Sorangeum cellosum (YP_001616706); M. xan., Myxococcus xanthus (YP_634103); B. thu., Bacillus thuringiensis (ZP_00738534.1). The number of C-terminal amino acids omitted from each protein sequence is indicated in parentheses at the end of each line in the alignment. The three conserved carboxylates are shown in black, the residues interacting with the triphosphate tail of the nucleotide bound in the nucleotidyl transfer site are shown in blue. The residues involved in the activation step are shown in red. Residues in yellow are conserved amino acids whose function was explained by the current BtTLP structures. (TIFF) [file pone.0067465.s001.tiff]

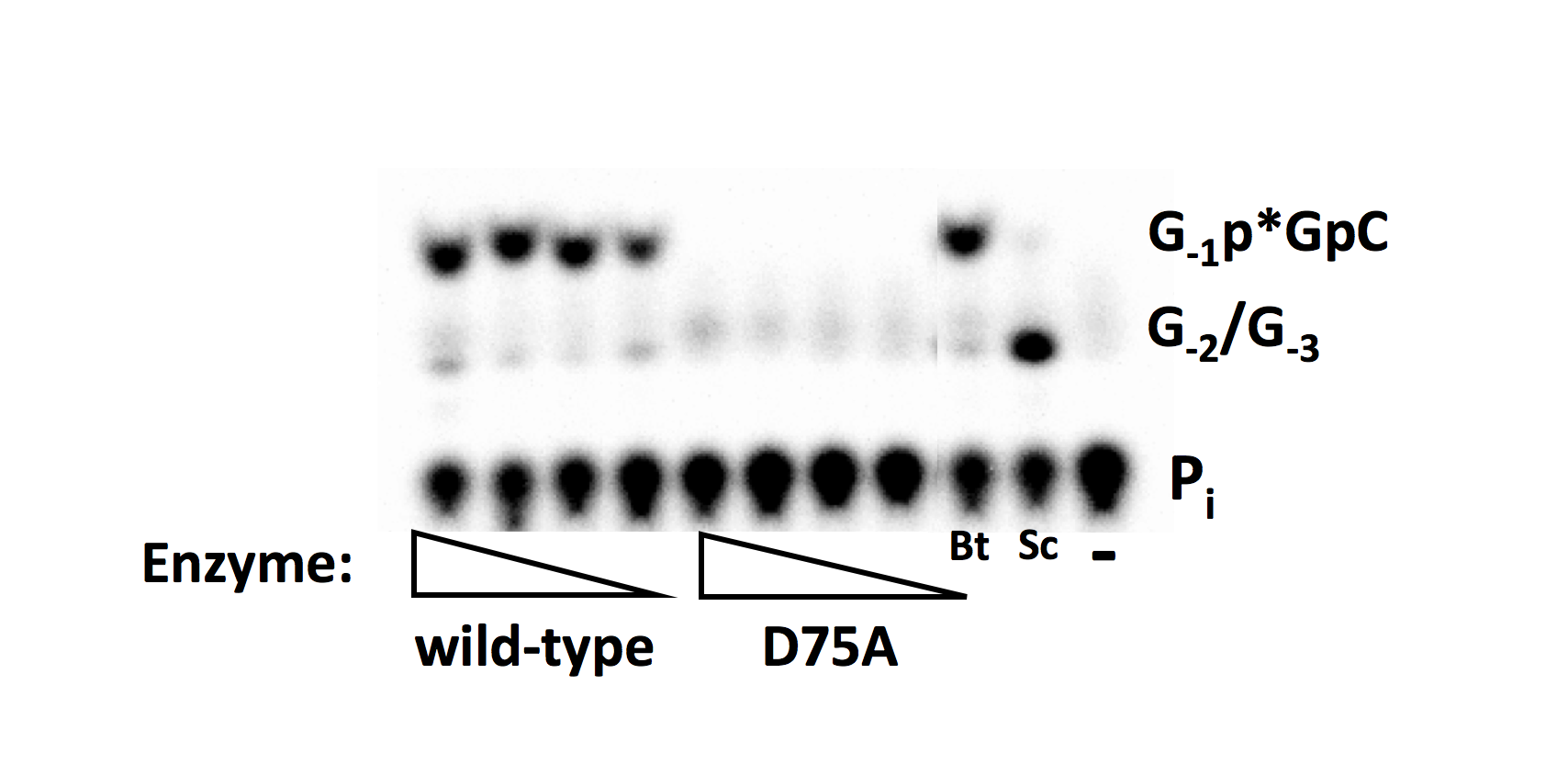

Supplement: Figure S2 — Mutating the metal-coordinating D75 in BtTLP to alanine results in a dramatically decreased enzymatic activity. Phosphatase protection assay of purified Bacillus thuringiensis TLP (BtTLP) (wild-type and D75A variant) for G−1 addition activity with 5′-32P-labeled tRNAHis. Addition of G−1/additional G-nucleotides results in production of phosphatase-resistant oligonucleotide products, as indicated to the right of the figure; in the absence of 3′-5′ addition activity, the labeled phosphate is removed by phosphatase treatment after the reaction and visualized as inorganic phosphate (Pi). Assays contained 5-fold dilutions of each purified enzyme (∼1–0.008 mg/ml). Lane Bt, control G−1 addition reaction with previously-purified BtTLP; lane Sc, control G−1 addition reaction with Saccharomyces cerevisiae Thg1 (ScThg1); lane -, buffer control. (TIF) [file pone.0067465.s002.tif]

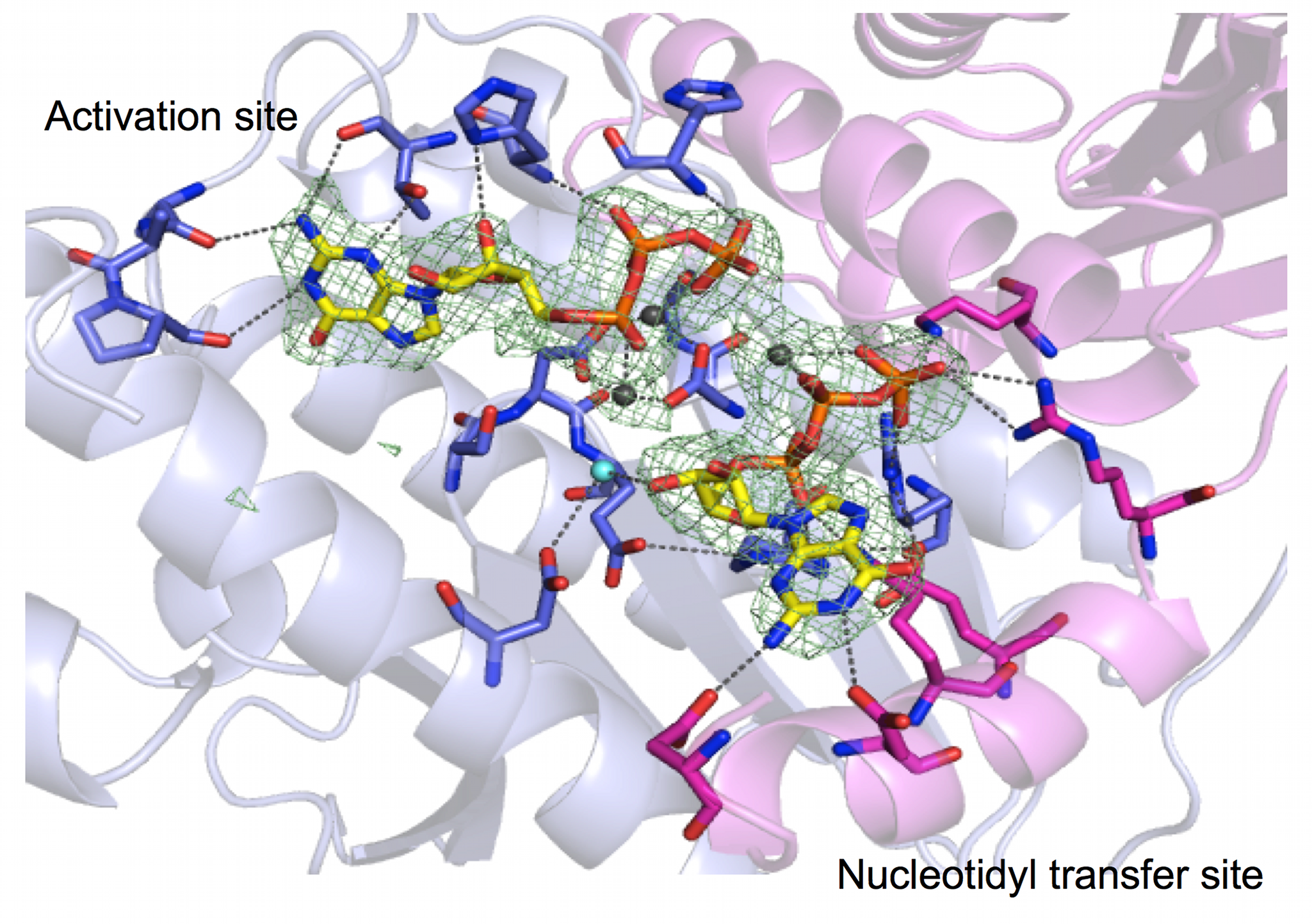

Supplement: Figure S3 — Close-up view of the activation and nucleotidyl transfer sites in the GTP-bound BtTLP model with overlaid Fo-Fc map (green mesh) contoured at 3 σ. (TIF) [file pone.0067465.s003.tif]

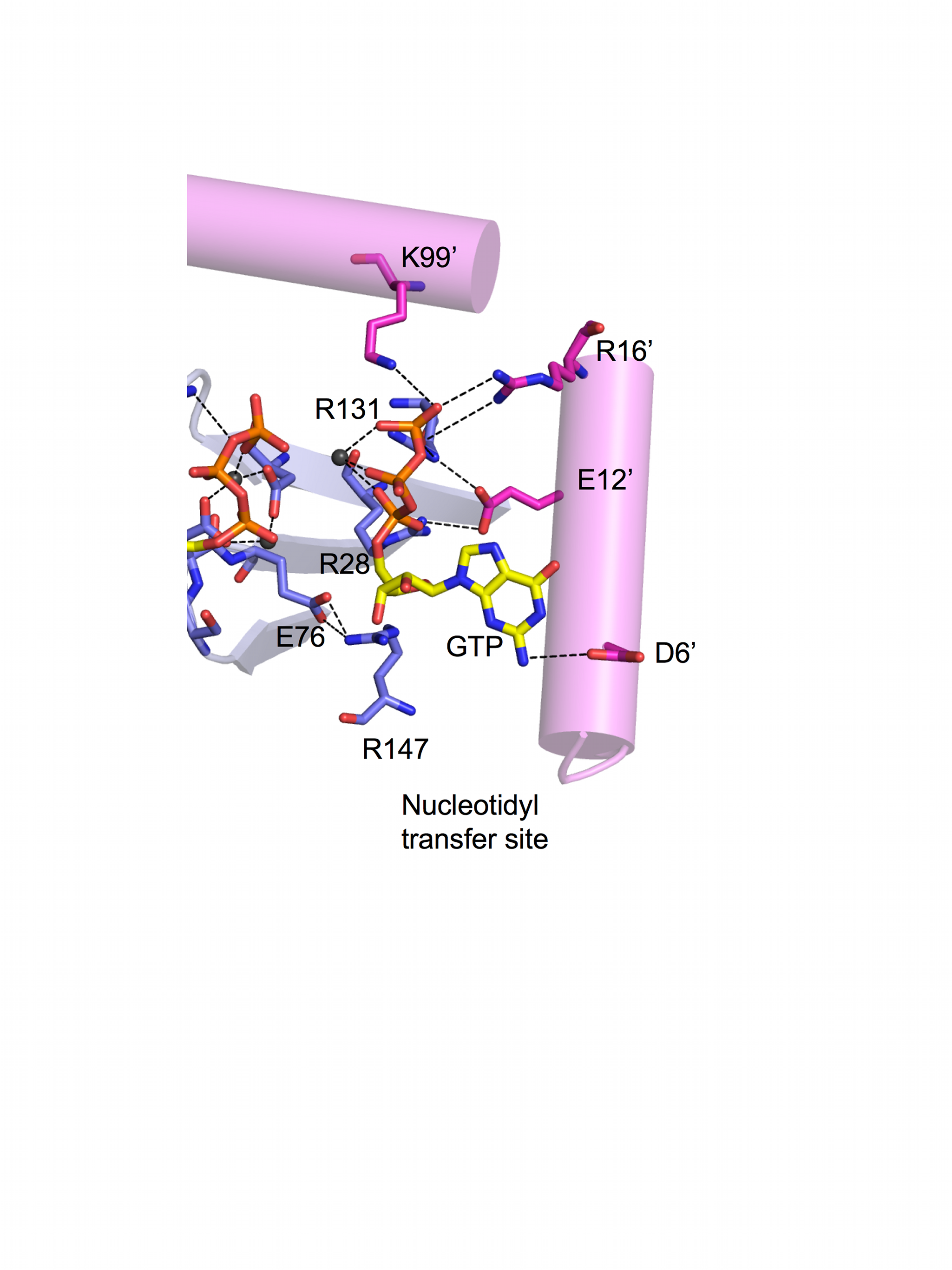

Supplement: Figure S4 — Close-up view of the BtTLP nucleotidyl transfer site and second GTP. The view was rotated compared to Figure 4A in order to display all enzyme-nucleotide interactions. The triphosphate moiety of the GTP in the activation site is seen on the left. (TIF) [file pone.0067465.s004.tif]
